# Dynamics and orientation selectivity in a cortical model of rodent V1 with excess bidirectional connections

**DOI:** 10.1038/s41598-019-40183-8

**Published:** 2019-03-04

**Authors:** Shrisha Rao, David Hansel, Carl van Vreeswijk

**Affiliations:** CNPP, CNRS UMR 8119, 45 Rue des Saints-Pères, 75270 Paris cedex 06, France

## Abstract

Recent experiments have revealed fine structure in cortical microcircuitry. In particular, bidirectional connections are more prevalent than expected by chance. Whether this fine structure affects cortical dynamics and function has not yet been studied. Here we investigate the effects of excess bidirectionality in a strongly recurrent network model of rodent V1. We show that reciprocal connections have only a very weak effect on orientation selectivity. We find that excess reciprocity between inhibitory neurons slows down the dynamics and strongly increases the Fano factor, while for reciprocal connections between excitatory and inhibitory neurons it has the opposite effect. In contrast, excess bidirectionality within the excitatory population has a minor effect on the neuronal dynamics. These results can be explained by an effective delayed neuronal self-coupling which stems from the fine structure. Our work suggests that excess bidirectionality between inhibitory neurons decreases the efficiency of feature encoding in cortex by reducing the signal to noise ratio. On the other hand it implies that the experimentally observed strong reciprocity between excitatory and inhibitory neurons improves the feature encoding.

## Introduction

Cortical neurons with similar functional properties have a high probability of being connected^1–7^. Experimental techniques that allow labeling and identifying different cell types has led to the discovery of patterns in cortical wiring at a scale finer than cortical columns. For instance, discrete subsets of neurons are more strongly interconnected than dictated by similarities in their functional properties: cortical networks embed motifs formed by strongly connected groups of neurons and groups of neurons receiving common feedforward input. As a result there are significantly more reciprocal connections between excitatory neurons than expected by chance. Likewise there is also an excess of other motifs involving groups of three or more highly interconnected excitatory neurons. This has been shown to be the case in layer 2/3^8^ and layer 5^9–11^. As for excitatory and fast spiking inhibitory neurons, the probability that their connections are reciprocal is close to one^1^. This raises the question: what are the contributions of such fine structures to cortical dynamics and fonction?

Previous theoretical studies investigating the dynamics of model cortical networks have assumed that the probabilities of connection are independent. In these networks, the probability of connection of a neuron A to a neuron B does not depend on the rest of the network graph, including whether there is a connection from neuron B to neuron A. For instance, a great deal of theoretical studies assume a directed Erdös-Rényi graph as the network architecture, in which the probability of connection depends solely on the neuronal type, excitatory or inhibitory, of the pre and postsynaptic neuron. It should be noted, however, that if the probability of connections are independent, the network has no fine structure. Thus these studies cannot shed light on the dependence of cortical dynamics and function on the fine structures in the connectivity.

Here we study the effect of excess bidirectionality in a model of layer 2/3 in rodent primary visual cortex (V1). The network is highly recurrent with strong synapses^12–14^. In the absence of excess bidirectionality the dynamics exhibits irregular spiking closely resembling experimental observations^15–17^ and the firing rates of the neurons depend on stimulus orientation. Using numerical simulations, we investigate the effects of introducing extra reciprocal connections on both the dynamics of the network and the selectivity properties of the neurons.

## Results

The network consists of two populations of neurons, one excitatory (E) and the other inhibitory (I). Each neuron is described by a single compartment conductance-based model. The connectivity of the network is random with the neurons in both populations receiving on average *K* excitatory and *K* inhibitory recurrent inputs. Excitatory and inhibitory neurons also receive feedforward inputs from, on average, ${K}_{\alpha }^{ff}(\alpha =E,I)$ randomly chosen excitatory L4 neurons. The responses of L4 neurons to elongated stimuli are orientation selective (OS) with uniformly distributed preferred orientations (POs) (see Methods).

**Figure 1:**
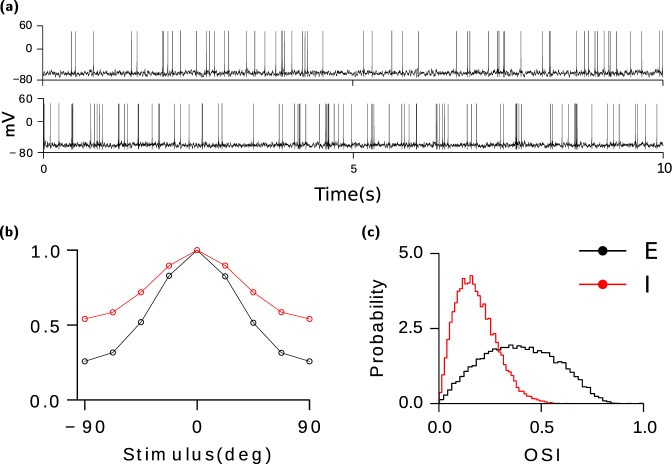
Activity in the network without excess bidirectionality. Other parameters are given in Methods section. (**a**) Sample voltage trace of cells (firing rates: top, E: 5.36 Hz, bottom, I: 8.7 Hz). (**b**) Population averaged tuning curves for both populations (E: black; I: Red). The tuning curves are normalized to the peak rate. (**c**) Distribution of orientation selectivity index (OSI) for excitatory (black) and inhibitory (red) neurons. Unlike in van Vreeswijk and Hansel^18^, the average number of feedforward inputs from layer 4 in excitatory and inhibitory neurons are different: ${K}_{ff}^{E}=100$, ${K}_{ff}^{I}=800$. Inhibitory neurons receive more but weaker feedforward inputs leading to less selectivity in their response.

Without excess bidirectionality, the probability, $P(i\to j)$, that neuron *i* connects to neuron *j* is $K/{N}_{A}$, where *N*_*A*_ $(A=E,I)$ is the number of neurons in the population to which neuron *i* belongs. The probability of a bidirectional connection, $P(i\to j\wedge j\to i)$, satisfies: $P(i\to j\wedge j\to i)=P(i\to j)P(j\to i)$. As previously shown^12–14^, if the interactions are strong the network automatically finds an operating point where total excitatory and inhibitory inputs to the neurons approximately cancel, *i*.*e*. *balance* each other. Thus, in the *balanced state* the net input to neurons consists of mean and fluctuations that are of the same magnitude as that of rheobase current (${\mathscr{O}}(Threshold)$). The temporal firing pattern of neurons in *balanced networks* is dominated by the fluctuations and is thus irregular (Fig. 1a).

The preferred orientations of the Layer 4 inputs arriving at a Layer 2/3 neuron are randomly distributed. Thus the total feed-forward input consists of a large component which is untuned to stimulus orientation and a tuned component whose amplitude is comparable to the neuronal threshold. Since the network is in the balanced state, the untuned component of the feed-forward is canceled by the average recurrent input. As a result, the net input in Layer 2/3 neurons as an untuned component, modulation with orientation and temporal fluctuations, all of which are comparable to the rheobase. Hence, neurons in the network exhibit orientation selectivity (Fig. 1b) and irregular firing^18^.

To change the amount of bidirectionality we rearrange the recurrent connectivity such that the probability, $P(A\to B\wedge B\to A)$, is equal to $pP(B\to A)$ without changing the in-degree distributions (see Methods). If $p > P(A\to B)$, the connectivity exhibits excess bidirectionality. Excess bidirectionality increases the number of loops embedded in the network connectivity which may effect both the spatial and temporal components of input fluctuations.

We study the effect of increasing the amount of bidirectionality for excitatory to excitatory (EE), inhibitory to inhibitory (II) and between excitatory and inhibitory (EI) connections. We first show that the network is still in the balanced state. Then we consider for each case the effect on the spike statistics and the tuning properties.

### Excess bidirectionality leads to non negligible effective self coupling

The average number of small loops in this network remains finite in the large *N*_*A*_ limit. For instance, a given neuron participates in pK loops of length two. These loops will give rise to an effective self-coupling of ${\mathscr{O}}(Threshold)$. While this does not effect the population average firing rates (see Supplementary Fig. S1) it can effect the rate distribution and temporal statistics. To see how loops in the network connectivity can contribute effects of ${\mathscr{O}}(Threshold)$, let us consider one excitatory neuron in the excitatory population. When p is not too large, loops of length two will dominate the effective self-coupling. If neuron i emits a spike at time t, this will increase the input to all the excitatory neurons it projects to by an amount ${J}_{EE}/\sqrt{K}$, which will on average elicit ${\xi }_{E}{J}_{EE}/\sqrt{K}$ extra spikes. Here ${\xi }_{E}$ is the average gain of the excitatory neurons. Since on average pK of these neurons project back to neuron i, the spike of neuron i at time t will result in an extra feedback input with some delay whose integral is given by $pK\,{\xi }_{E}({J}_{EE}/\sqrt{K})\,({J}_{EE}/\sqrt{K})=pK{\xi }_{E}{J}_{EE}^{2}$.

Similarly, with excess bidirectionality between inhibitory neurons, the effective self coupling has an integral $pK{\xi }_{I}{J}_{II}^{2}$, while for excess bidirectionality between excitatory and inhibitory neurons the integral of the effective self coupling is $pK{\xi }_{I}{J}_{EI}{J}_{IE}$ for the excitatory neurons and $pK{\xi }_{E}{J}_{EI}{J}_{IE}$ for the inhibitory population. Here we have assumed the network to be sparse. When the network is dense i.e. when *K* is comparable to *N*_*E*_ and *N*_*I*_, excess bidirectionality has the same effect. Analysis of the dense network, however, is more involved.

Although the effective self-coupling in the input which is induced by excess bidirectionality is non negligible, it does not destroy the balanced state because it is of the same order as the other components of the net input, namely ${\mathscr{O}}(Threshold)$. It is, however, sufficiently strong to potentially affect the spike statistics and tuning properties.

### E-to-E bidirectionality has negligible effect on spike statistics

**Figure 2:**
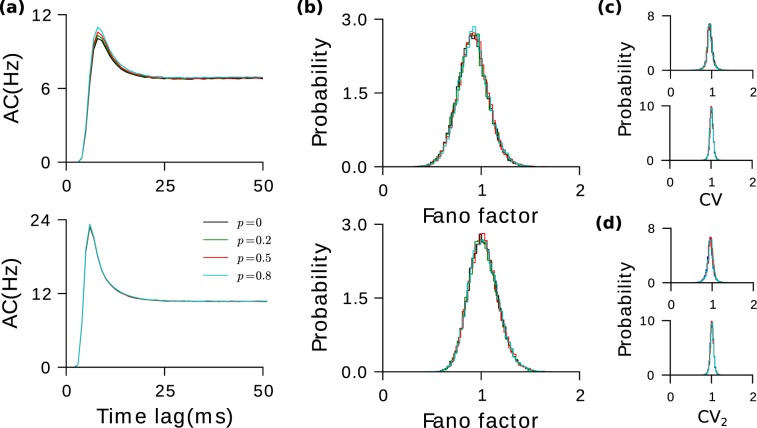
Bidirectionality in E-to-E has negligible effect on spiking irregularity. (**a**) Population averaged autocorrelation functions for excitatory and inhibitory populations for different values of *p*. (**b**) Fano factor distributions different values of *p*. (**c**) Distribution of *CV*. (**d**) Distribution of *CV*_2_ (see Methods). In all subfigures the top panel is for the excitatory population and the bottom one is for the inhibitory population.

Excess E-to-E bidirectionality should lead to delayed positive self-coupling which may give rise to temporal correlations in the fluctuations. Surprisingly, we found that in our numerical simulations, introducing excess bidirectionality in E-to-E connections does not lead to any observable changes in the statistics of the fluctuations in the network. For each population, the spike time autocorrelation (AC) function was computed for the neurons and averaged. Figure 2a displays for different values of *p* the averaged autocorrelation for the excitatory and inhibitory population. There is no perceptible change as *p* is increased. The Fano factor distributions (Fig. 2b) of the neurons in the two populations remain unchanged and so do the distributions of the coefficients of variation, *CV* (Fig. 2c) and *CV*_2_ (Fig. 2d).

### I-to-I bidirectionality induces positive serial correlations in inter-spike intervals

As for *EE* excess bidirectionality, I-to-I bidirectionality gives rise to a positive effective self-coupling. This self coupling is due an effective self-disinhibition. Consider neuron (*i*, *I*). After it has fired a spike, it hyperpolarizes its postsynaptic inhibitory neurons by a small amount, slightly decreasing their probability of spiking. Since a fraction *p* of those neurons project back to neuron (*i*, *I*), this decreases the amount of inhibition the latter receives, thereby increasing the probability that it will spike again.

**Figure 3:**
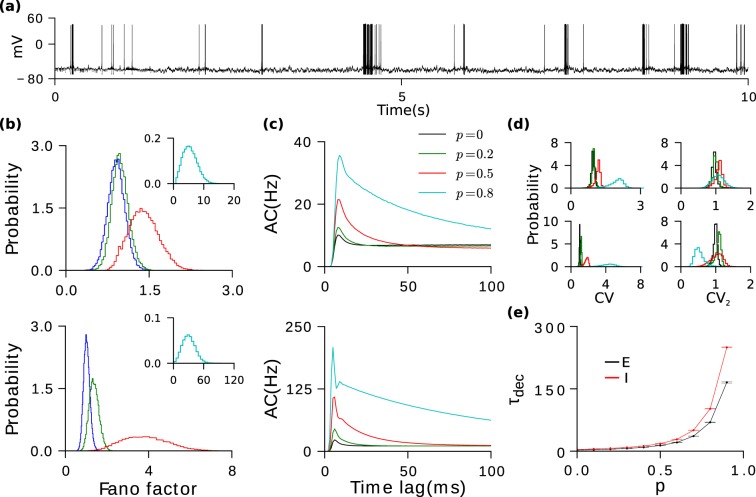
Bidirectionality in I-to-I slows down fluctuations and increases response variability. (**a**) Example voltage trace of an inhibitory cell for p = 0.8 (firing rate: 8.8 Hz). Dependence on *p* of the Fano factor (**b**), population averaged autocorrelation functions (**c**), *CV* and *CV*_2_ in (**d**). Top panels: Excitatory population. Bottom panesl: Inhibitory population. (**e**) Decorrelation time (see Methods) of the network activity as a function of *p*.

Our numerical simulations show that this significantly affects the dynamics. The inter-spike intervals (*ISIs*) of inhibitory neurons become positively correlated and they now have a tendency to fire in bursts (Fig. 3a). This is reflected by an increase in their average *CV* with hardly any change in their *CV*_2_ (Fig. 3d) for *p* as large as 0.5. Such serial correlations in the spike trains also lead to increasing trial-to-trial variability (Fig. 3b). Furthermore, the serial correlations affect the spike AC. It now decays to its asymptotic value with a time constant that increases with *p* (Fig. 3c and Supplementary Fig. S3). The positive correlations in the inhibitory ISIs also produce slow fluctuations in the inhibitory feedback to the excitatory neurons. This leads to positive correlations between excitatory ISIs yielding in similar but smaller changes in the dynamics of the excitatory population (Fig. 3b–e).

### E-I bidirectionality introduces negative auto-correlations

There is strong experimental evidence that the probability of excess bidirectionality in the connectivity of PV^+^ interneurons and pyramidal cells is close to one^1^. Using similar arguments as before, it is clear that such E-I bidirectionality produces an effective negative self-coupling for neurons in both populations. This leads to negative serial correlations in the ISIs.

**Figure 4:**
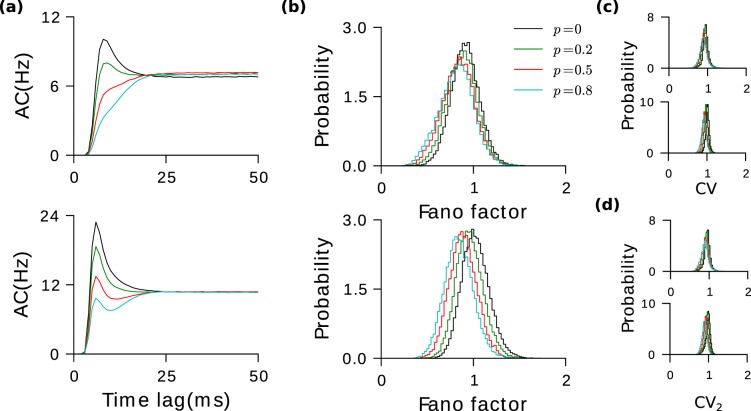
Bidirectionality in E-to-I connections leads to rapid decorelation and reduced response variability. (**a**) Population averaged autocorrelation functions for different values of *p*. (**b**) Average Fano factor decreases with *p*. The distributions of *CV* (**c**) and *CV*_2_ (**d**) have negiligible dependance on *p*. Top panels: Excitatory neurons. Bottom panels: Inhibitory neurons.

In our numerical simulations, the population averaged AC functions now show a distinct negative undershoot before they converge to their asymptotic values (Fig. 4a). The magnitude of the undershoot increases with *p* while its duration is unchanged. The distribution of *CV* hardly changes with *p* indicating that on the time scale of the neuronal input integration the input statistics has a very weak dependence on *p* (Fig. 4c). The changes in *CV*_2_ are also small because correlations between consecutive ISIs are weak (Fig. 4d). There are also negative correlations between more distant ISIs. Because these accumulate in the Fano factor (Eq. 16), its reduction is more significant (Fig. 4b).

### Tuning properties are qualitatively preserved in the presence of bidirectional connectivity

The positive self-coupling for bidirectionality within a population *α* (E or I) increases the modulation with stimulus orientation of the time averaged net input into neurons. This suggests an increase in the orientation selectivity index (*OSI*, see Methods) of neurons in population *α*.

**Figure 5:**
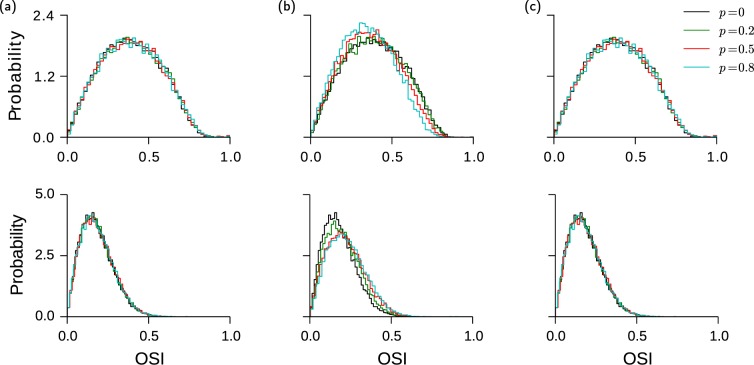
Bidirectionality has a weak effect on feature selectivity. (**a** and **c**) Excess bidirectionality within the excitatory population (**a**) and between excitatory and inhibitory populations (**c**) have no effect on the selectivity of excitatory (top) and inhibitory (bottom) neurons. Excess bidirectionality between the inhibitory neurons slightly decreases the selectivity of excitatory neurons (**b**, top) while it slightly increases the selectivity in inhibitory population (**b**, bottom).

The amount of sharpening depends on the magnitude of the effective positive self-coupling, which can be fairly large when I-to-I connections are bidirectional. In our simulations, excess reciprocity in I-to-I connections has a noticeable but nevertheless small effect on the degree of tuning of excitatory as well as inhibitory neurons. When excess I-to-I reciprocal probability is increased from 0 to 0.8, the mean *OSI* increases by 24% for the inhibitory neurons. In contrast, for the excitatory population it decreases by 11% (Fig. 5b).

The activity becomes more butsty with increasing *p*, which results in an increase in the temporal fluctuations in the input. This leads to a reduction in the neuronal gain. In the excitatory population this broadens the tuning curves. In the inhibitory neurons the situation is more subtle. The reduction in their gain is comparable to the one of the excitatory neurons but their self-coupling tends to also increase the selectivity of the time averaged input. In our simulations, the latter dominates and the tuning of the inhibitory neurons sharpens. (see Supplementary Fig. S4).

As was the case for network dynamics, our simulations show that E-to-E bidirectionality has negligible effects on the neuronal tuning properties even when *p* is close to one. At bidirectional probability as high as $p=0.8$, there is no noticeable change in the *OSI* distributions in both populations (Fig. 5a,c).

Excess bidirectionality between excitatory and inhibitory neurons leads to negative self-coupling, suggesting a decrease in the mean *OSI* of both populations. Since this excess has a moderate effect on the dynamics, one would expect a moderate change in orientation selectivity. However, simulations show no noticeable change in the *OSI* distributions when *p* is increased. The reason for this is that negative serial correlations in the spike statistics reduce the fluctuations in the inputs leading to a larger gain. This increase in gain counterbalances the decrease in the modulation of the time averaged inputs due to the effective negative self-coupling.

## Discussion

Previous theoretical studies of cortical networks have mostly considered connectivities described by a directed Erdös-Rényi like graph where reciprocal connections occur by chance. To our knowledge, this is the first study which investigates the impact on cortical dynamics and functional properties of fine structure in the connectivity. We considered a conductance-based model of layer 2/3 of rodent V1 in which neuronal interactions are strong. We studied the effect of excess bidirectionality in this model. Excess bidirectionality results in an effective self-coupling of ${\mathscr{O}}(Threshold)$. Thus, although the network with excess reciprocal connections operates in the balanced state similar to when reciprocity occurs by chance, the neuronal self-coupling can affect the network activity.

In our simulations, we found that excess bidirectional connections in the excitatory population have negligible effect on the dynamics and function. Extra reciprocal connections between inhibitory neurons increase the selectivity of inhibitory cells but decrease that of excitatory ones. They slow down the temporal fluctuations in the activity of the inhibitory and, to a lesser extent, the excitatory population. This results in spike autocorrelations that decay slowly. In contrast, excess bidirectionality between excitatory and inhibitory neurons gives rise to an undershoot in the spike autocorrelations but negligibly affects orientation selectivity.

In our model, excitatory interactions are weaker that in inhibitory ones. Moreover, since the firing rate of the excitatory neurons is smaller than for the inhibitory neurons, the gain of the former is smaller than that of the latter. As a result, excess bidirectionality between the excitatory neurons has a much weaker effect that between inhibitory neurons. In principle, the effect of excess reciprocity in the excitatory population can be enhanced. For example, decreasing the number of recurrent EE connections and increasing their synaptic efficacy by the same factor could make the effect of E-to-E bidirectionality stronger. Alternatively, the gain of the excitatory neurons can be enhanced by changing the parameters so that their firing rate is increased. Both scenarios are biologically plausible. Whether there is a reasonable mechanism that leads to an appreciable effect of excess bidirectionality between excitatory neurons is an open question.

The effects of symmetric connectivity on the network dynamics and emergence of multistability has been studied in diluted Spin Glass (SG) models^19–21^. Although, a direct analogy between our model and SG models is unwarranted (for instance, Dale’s law is violated in the latter model), the analytical results from those models may provide insights for interpreting our results. A connectivity matrix with bidirectional probability of one is a symmetric matrix. With symmetric connectivity matrices, it has been shown that SG phase is stable in the low temperature regime. The SG phase is characterized by non-decaying temporal correlations and dependence on initial conditions. In presence of asymmetry the SG phase is stable only at zero temperature. For finite temperature, the temporal correlations decay to zero. Correlation time increases with the level of symmetry *p*, in this dependence is well approximated by a power law in 1 − *p*. In our model, the decorrelation time shows approximately similar power law behavior (see Supplementary Material; Fig. S2a).

In balanced networks, the inhibitory population is the main source of fluctuations. The variance of input fluctuations could be seen as a temperature-like quantity. With fast synapses the variance of the input fluctuations is large, i.e. the “temperature” is high. Slow synapses have a filtering effect. Input fluctuations now decorrelate over the time scale of synaptic time constant, which has the consequence of reducing the variance. But, since the network is operating in the balanced regime, the variance remains ${\mathscr{O}}(Threshold)$. Hence, although slower synapses play the role of lowering the “temperature”, the temperature stays finite. In general, our result is consistent with the theoretical prediction that the SG phase is unstable at finite temperature and asymmetry, i.e. there is no multistability (see Supplementary Material). Slow synapses and excess bidrectionality in the inhibitory population leads to increase in Fano factor (Fig. S2b). As a consequence, the measurement time window now required to achieve the same error bounds in estimating firing rates with slow synapses is increased compared to that with fast synapses. On the scale of a few hundreds of seconds, this can be effectively regarded as multistability.

How could excess bidirectionality affect biological functions? Since only excitatory neurons project to other cortical areas, we only need to consider the effect of excess bidrectionality on the excitatory population. For example, let us consider the ability of an “optimal observer” to estimate the orientation of a stimulus in rodent V1. Excess I-to-I bidirectionality increases the Fano factor of both populations, which leads to a reduction in the decoding accuracy. In addition, it tends to decrease the average OSI of the excitatory neurons. For a one dimensional stimulus feature such as orientation, the Fisher information is inversely proportional to the tuning width^22^. These two factors together imply a reduction of decoding accuracy. On the other hand, bidirectionality between excitatory and inhibitory neurons decreases the Fano factor while it hardly affects the tuning properties. This suggests that EI bidirectionality increases the decoding accuracy. A quantitative answer to the question of how connection reciprocity affects coding necessitates a systematic study, which is not the focus here.

## Methods

### Model of rodent L2/3 with excess bidirectionality

Neurons in the L2/3 of rodents show strong orientation selectivity (OS) already at eye opening. They are arranged in a salt and pepper fashion so that each integrated inputs from neurons of all preferred orientations(PO) (i.e. the rodent V1 lacks an orientation map or functional architecture). To investigate the effect of bidirectionality on the spiking irregularity and functional properties of the cortex, we used a modified version of a conductance based spiking model of rodent L2/3 developed in Hansel and Vreeswijk^18^. They showed that strong OS does not require a functional architecture, provided that the cortex is operating in the balanced regime. Connection probability between neurons was fixed such that each neuron neuron recieved on average *K* synaptic inputs. L4 neurons were assumed to be OS and L2/3 neurons received feedforward inputs from randomly selected L4 neurons with different POs. Hence the total input that each L2/3 neurons receives has a large untuned component and a comparably weak tuned part. In the model, the recurrent dynamics of the network is such that the total inhibitory and excitatory currents cancel each other. Hence, the large untuned component is dynamically suppressed. The tuned component which is ${\mathscr{O}}(Threshold)$ is now revealed rendering the neurons in the network selective to orientation of the external stimulus.

### Single neuron dynamics

The single neuron dynamics are described by an one compartment conductance based model with sodium and potassium currents responsible for spike generation^23^. The membrane potential ${V}_{i}^{A}$ of a neuron *i* in population *A* is described by,

1 $${C}_{m}\frac{d{V}_{i}^{A}}{dt}=-\,{I}_{L,i}^{A}-{I}_{Na,i}^{A}-{I}_{K,i}^{A}-{I}_{adapt,i}^{A}+{I}_{rec,i}^{A}+{I}_{ff,i}^{A}+{I}_{b,i}^{A}$$

where *C*_*m*_ is the membrane capacitance, *I*_*L*_ is the leak current given by ${g}_{L}^{A}({V}_{i}^{A}-{V}_{L})$. The voltage dependent sodium and potassium currents are given by ${I}_{Na,i}^{A}={g}_{Na}^{A}{m}_{\infty }^{3}h({V}_{i}^{A}-{V}_{Na})$ and ${I}_{K,i}^{A}={g}_{K}^{A}{n}^{4}({V}_{i}^{A}-{V}_{K})$. We assume that the activation of the sodium current is instantaneous, ${m}_{\infty }={\alpha }_{m}(V)/({\alpha }_{m}(V)+{\beta }_{m}(V))$. The gating variable *h* and *n* have the following kinetics^24^

2 $$\frac{dx}{dt}={\alpha }_{x}(V)\,(1-x)-{\beta }_{x}(V)\,x$$

The excitatory neurons have an additional adaptation current ${I}_{adapt,i}^{E}={g}_{adapt}^{E}z({V}_{i}^{E}-{V}_{K})$. The dynamics of the gating variable *z* is

3 $$\frac{dz}{dt}=\frac{{z}_{\infty }(V)-z}{{\tau }_{adapt}}\,{\rm{where}}\,{z}_{\infty }(V)=\frac{1}{1+\exp (\,-\,0.7(V+30))}$$

The external input to a neuron has three components: the recurrent synaptic input from within layer 2/3, ${I}_{rec,i}^{A}$, the feedforward input from layer 4 into layer 2/3 ${I}_{ff,i}^{A}={g}_{ff,i}^{A}(\theta ,t)\,({V}_{i}^{A}-{V}_{E})$ and, a background term ${I}_{b,i}^{A}$, which accounts for the input from other cortical regions. Given the connectivity matrix ${C}_{ij}^{AB}=0,1$, the recurrent current into neuron (*i*, *A*) due *k* spikes emitted by a neuron (*j*, *B*) at times ${t}_{j,k}^{B}$ is

4 $${I}_{rec,i}^{A}=-\,\sum _{B}\,{g}_{i}^{AB}(t)\,[\rho ({V}_{i}^{A}-{V}_{B})+(1-\rho )\,({V}_{L}-{V}_{B})]$$

5 $${g}_{i}^{AB}(t)=\frac{{\bar{g}}^{AB}}{{\tau }_{syn}}\,\sum _{j}\,{C}_{ij}^{AB}\,\sum _{k}\,\exp [\,-\,(t-{t}_{j,k}^{B})/{\tau }_{syn}]$$

The background input is

6 $${I}_{b,i}^{A}=-\,\sum _{B}\,{g}_{b,i}^{A}(t)\,[\rho ({V}_{i}^{A}-{V}_{E})+(1-\rho )\,({V}_{L}-{V}_{E})]$$

7 $${g}_{b,i}^{A}(t)={\bar{g}}_{b}^{A}K\,({R}_{b}^{A}+\sqrt{\frac{{R}_{b}^{A}}{K}}{\eta }_{b,i}^{A}(t))$$

where ${\eta }_{b,i}^{A}(t)$ is a zero mean Gaussian noise with temporal correlation, $\langle {\eta }_{b,i}^{A}(t){\eta }_{b,i}^{A}(t^{\prime} )\rangle =\exp (\,-\,|t-t^{\prime} |)/2{\tau }_{syn}$. The feedforward input into neuron (*i*, *A*) for an external stimulus orientation *θ* of contrast *C* is given by ${I}_{ff,i}^{A}(\theta ,t)$ = $-\,{\sum }_{B}\,{g}_{ff,i}^{A}(t)\,[\rho ({V}_{i}^{A}-{V}_{E})+(1-\rho )\,({V}_{L}-{V}_{E})]$ where

8 $${g}_{ff,i}^{A}(\theta ,t)=\frac{{\bar{g}}_{ff}^{A}}{{\tau }_{syn}}\,{\int }_{-\infty }^{t}\,({R}_{i,tot}^{A}(\theta ,t^{\prime} )+\sqrt{{R}_{i,tot}^{A}(\theta ,t^{\prime} )}{\eta }_{i}^{A}(t)){e}^{-(t^{\prime} -t)/{\tau }_{syn}}dt^{\prime} $$

with

9 $${R}_{i,tot}^{A}(\theta ,t)={c}_{ff}^{A}K\,[{R}_{0}^{ff}+{R}_{1}^{ff}(C)]+\sqrt{{c}_{ff}^{A}K}\,({x}_{i}^{A}+{R}_{1}^{ff}(C)\,[{x}_{i}^{A}+{\xi }_{A}{z}_{1,i}^{A}\,\cos \,2(\theta -{{\rm{\Delta }}}_{i}^{A})])$$

where, ${\xi }_{A}$ depends on the tuning strength of layer 4. The random variables ${x}_{i}^{A}$, ${z}_{i}^{A}$, ${{\rm{\Delta }}}_{i}^{A}$ are independently drawn from, a standard normal distribution, $z{e}^{-\frac{{z}^{2}}{2}}$ and, an uniform distribution on the interval [0, *π*] respectively. ${R}_{0}^{ff}$ is baseline activity of layer 4 neurons in the absence of a stimulus and ${R}_{1}^{ff}={R}_{1}^{ff}\,{\mathrm{log}}_{10}(C+1)$ is the amplitude of th layer 4 response to stimulus. The strength of recurrent synaptic interactions were scaled as ${\overline{g}}_{AB}=\frac{{G}_{AB}}{\sqrt{K}}$, and the feedforward input as ${\overline{g}}_{ff}^{A}=\frac{{G}_{ff}^{A}}{{c}_{ff}^{A}\sqrt{K}}$ where ${c}_{ff}^{A}=\frac{{K}_{ff}^{A}}{K}$.

### Parameters used

The average conductances and leak currents are compatible with experimental reports^25^. We set, ${g}_{Na}=100\,mS/c{m}^{2}$, ${V}_{Na}=55\,mV$, ${g}_{K}=40\,mS/c{m}^{2}$, ${V}_{K}=-\,80\,mV$, ${V}_{L}=-\,65\,mV$, ${C}_{m}=1\,\mu F/c{m}^{2}$, ${g}_{L}=0.1\,mS/c{m}^{2}$. Only excitatory neurons had adaptation current with ${g}_{adapt}=0.5\,mS/c{m}^{2}$ and ${\tau }_{adapt}=60\,ms$. The synaptic time constant ${\tau }_{syn}$ was set to 3 *ms*. ${G}_{EE}=0.15$, ${G}_{IE}=0.45$, ${G}_{EI}=2.0$, ${G}_{II}=3\,ms\,mS/c{m}^{2}$. ${\xi }_{A}=0.8$, $C=100$, ${R}_{0}^{ff}=2\,Hz$, ${R}_{1}^{ff}=20\,Hz$. $K=500$^26^, ${K}_{ff}^{E}=100$, ${K}_{ff}^{I}=800$.

### Generating excess bidirectionality in the connectivity matrix

To generate the connectivity matrix with an excess bidirectionality of *p*, a neuron *i* from population *A* and neuron *j* from population *B* were connected reciprocally with a probability of ${p}_{ij}^{AB}=p\frac{K}{{N}_{B}}+(1-p)\frac{{K}^{2}}{{N}_{B}^{2}}$. Unidirectional connections were made with a probability ${p}_{ij}^{AB}=(1-p)\frac{K}{{N}_{B}}(1-\frac{K}{{N}_{B}})$. This gives a connectivity matrix with each neuron receiving *K* inputs on average with *pK* number of bidirectional connections. Whereas, a random network has $\frac{{K}^{2}}{N}$ bidirectional connections on average.

### Orientation selectivity index (OSI)

The selectivity of a neuron that has a firing rate *r*(*θ*_*k*_) has an OSI given by $\frac{|z|}{{\sum }_{k}\,r({\theta }_{k})}$ where $z={\sum }_{k}\,r({\theta }_{k})\,\exp (2i{\theta }_{k})$. A broadly tuned neuron has an OSI close to zero and neurons which are more selective have an OSI closer to one.

### Fano factor

Given the spike count *N*_*k*_ of a neuron in trial *k*, Fano factor (FF) of that neuron is defined as,

10 $$FF=\frac{{\langle {({N}_{k}-\overline{N})}^{2}\rangle }_{k}}{\overline{N}},\,\overline{N}={\langle {N}_{k}\rangle }_{k}$$

where ${\langle \cdot \rangle }_{k}$ is the average over all trials. We repeated the simulation with different initial conditions while keeping the input stimulus fixed. The Fano factor was then determined for all neurons by computing the mean spike count and spike count variance upon repeated stimulus presentation over hundred simulated trials.

### Autocorrelation (AC)

Given a spike train $S(t)={\sum }_{k}\,\delta (t-{t}^{k})$, the autocorrelation function is defined as,

11 $$C(\tau )={\langle S(t)S(t+\tau )\rangle }_{t},$$

where ${\langle \cdot \rangle }_{t}$ is the average over time. We binned the spike train in Δ$t=1\,ms$ bins. Let the spike count in the *n*^*th*^ bin be *N*_*i*_(*n*). The population averaged autocorrelation function is defined as,

12 $$AC(\tau )={[\frac{{\langle {N}_{i}(t){N}_{i}(t+\tau )\rangle }_{t}}{{\rm{\Delta }}tT}]}_{i}$$

where $\tau =n{\rm{\Delta }}t$, $t=m{\rm{\Delta }}t$, *T* is the duration of simulation in seconds, and ${[\cdot ]}_{i}$ is the average over the population. The peak at zero was removed and the AC normalization is such that at long time lags the AC function of individual neurons converge to their respective mean activity squared. $(AC-{r}^{2})$ was plotted vs time on a loglog plot and the slope of the linear region was estimated by linear regression. This slope is considered to be the decay time of AC(${\tau }_{dec}$). Where, *r*_*i*_ is the mean firing rate of the *i*^*th*^ neuron. In the figures, *AC* was normalized such that it converges to the mean activity.

### Coefficient of variation (*CV* and *CV*_2_)

*CV* is the ratio of the standard deviation and mean of the spike *ISI*s. Given a spike train with *N* spikes occurring at times *t*_*i*_, the *ISI*s are given by,

13 $${\rm{\Delta }}{t}_{i}={t}_{i}-{t}_{i-1}$$

and the *CV* of the *ISI*s is defined as:

14 $$CV=\frac{\sqrt{{\langle {({\rm{\Delta }}{t}_{i}-\overline{{\rm{\Delta }}t})}^{2}\rangle }_{i}}}{\overline{{\rm{\Delta }}t}},\,\overline{{\rm{\Delta }}t}={\langle {\rm{\Delta }}{t}_{i}\rangle }_{i}$$

For a renewal process, FF is given by,

15 $$FF=C{V}^{2}$$

and for a stationary non-renewal process,

16 $$FF=C{V}^{2}(1+2\,\sum _{i}\,SR{C}_{i})$$

where *SRC*_*i*_ is the Spearman rank order correlation coefficient of order *i*. Each ISI was replaced by its rank among all ISIs. The rank of *ISI*_*k*_ is *r*_*k*_ if there are exactly *r*_*k*_ − 1 smaller intervals. *SRC*_1_ is the correlation between the ranks of adjacent ISIs. *SRC*_*i*_ is the correlation between the ranks of pairs of intervals, $(IS{I}_{k},IS{I}_{k-i})$, which are separated by *i* − 1 intervals. It is a measure of serial correlations in the spike trains. Positive serial correlations increase FF and negative serial correlations reduce FF. If a regular spike train has a slowly modulated firing rate, the CV obtained will be high even though the spike train is regular. To overcome this problem another measure, *CV*_2_, is usually adopted to quantify the intrinsic variability^16^. *CV*_2_ for the spike train is defined as:

17 $$C{V}_{2}={\langle 2\frac{|{\rm{\Delta }}{t}_{i+1}-{\rm{\Delta }}{t}_{i}|}{{\rm{\Delta }}{t}_{i+1}+{\rm{\Delta }}{t}_{i}}\rangle }_{i}$$

where ${\langle \cdot \rangle }_{i}$ stands for averaging over all the *N* spikes.

## Supplementary information

Suplementary material: Dynamics and orientation selectivity in a cortical model of rodent V1 with excess bidirectional connections
