# Supplementary material for: Dynamics and orientation selectivity in a cortical model of rodent V1 with excess bidirectional connections
Source: Sci Rep. 2019 Mar 4;9:3334. doi: 10.1038/s41598-019-40183-8 (PMC6399237; doi:10.1038/s41598-019-40183-8)
Supplement: Supplementary file 1 — Suplementary material: Dynamics and orientation selectivity in a cortical model of rodent V1 with excess bidirectional connections [file 41598_2019_40183_MOESM1_ESM.pdf]

Suplementary material:

Dynamics and orientation selectivity in a cortical model of rodent V1 with excess bidirectional connections

Shrisha Rao[1], **David Hansel**[1,*,+], **and Carl van Vreeswijk**[1,+]

[1]CNPP, CNRS UMR 8119, 45 Rue des Saints-Pères, 75270 Paris cedex 06
[*]Corresponding author: david.hansel@univ-paris5.fr
[+]Shared last authorship

Effects of slow synapses on stationary dynamics and transient responses in networks with excess I-to-I bidirectionality

The results in the paper are derived from simulations of networks in which all synaptic time constants are short (3ms). However, cortical networks have a significant number of slow synapses (e.g. $GABA_B$ and NMDA). How does the presence of slow synapses in addition to excess bidirectionality effect the input fluctuations? To answer this question, we performed simulations where we increase the time constants of both feedforward and recurrent synapses together. They show that the decorrelation time increases with synaptic time constant (Fig. S2). For small p, the decorrelation times scale linearly with synaptic time constants (Fig. S2a). For larger p, strong supra-linear deviations are apparent. When p approaches one, the decorrelation time increases dramatically.

Slower synaptic time constants lead to a decrease in temporal fluctuations and hence an increase in the gain of the neurons resulting in an amplification of the self-coupling. If the effective self-coupling is sufficiently positive, the network could become multistable. To check whether this is the case in our model, we performed simulations with a large number of randomly chosen initial conditions. We observed that after a transient, the network always converged to a state in which the firing rates of the individual neurons are the same. Thus, the network dynamics is not multistable. To investigate the dependence of this convergence on the synaptic time constant, we computed spike counts of individual neurons in time windows of different length. We then calculated the Spearman coefficient, ρ, of the spike count for pairs of different initial conditions as a function of the window size, T . As is the case for the decorrelation time, for p not close go 1, the time it takes for the network to reach the steady state scales increases linearly with the synaptic time constant, τ_{syn}., When p approaches one, this time increases supralinearly with τ_{syn} and becomes extremely large (Fig. S2c).

Supplementary figures

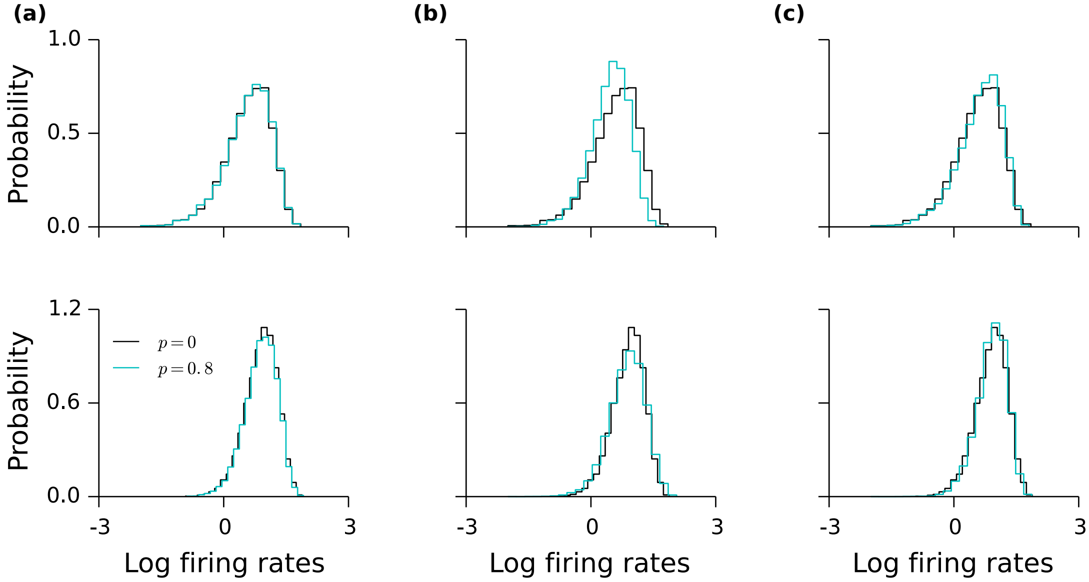

Figure S1. Firing rate distributions in absence of feature selectivity in the feedforward input with excess bidirectionality in E-to-E (a), I-to-I (b) and E-to-I (c) connections. Top: Excitatory neurons. Bottom: Inhibitory neurons.

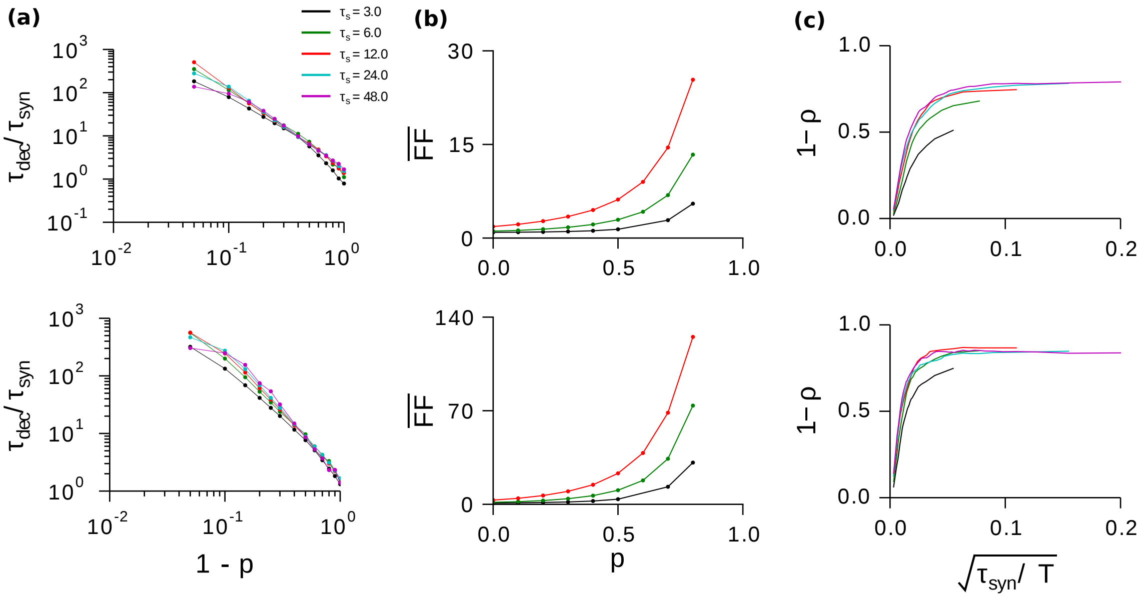

Figure S2. **Effect of the synaptic time constant on the slowing of the dynamics with I-to-I bidirectionality.** a: In the range $p = 0 - 0.8$, the estimated decorrelation time is proportional to τ_{syn} and to $(1-p)^{\alpha}$ with $\alpha = 2$. Substantial deviations from this are observed for $p > 0.8$ possibly due to the difficulty of estimating the time constant when the dynamics is extremely slow. b: The population averaged Fano factor increases with p and with τ_{syn}. The rate of convergence to the steady state firing rates of excitatory neurons for two different initial conditions quantified by computing the Spearman coefficient (ρ) as a function of observation window (T) for $p = 0$ (top) and $p = 0.90$ (bottom).

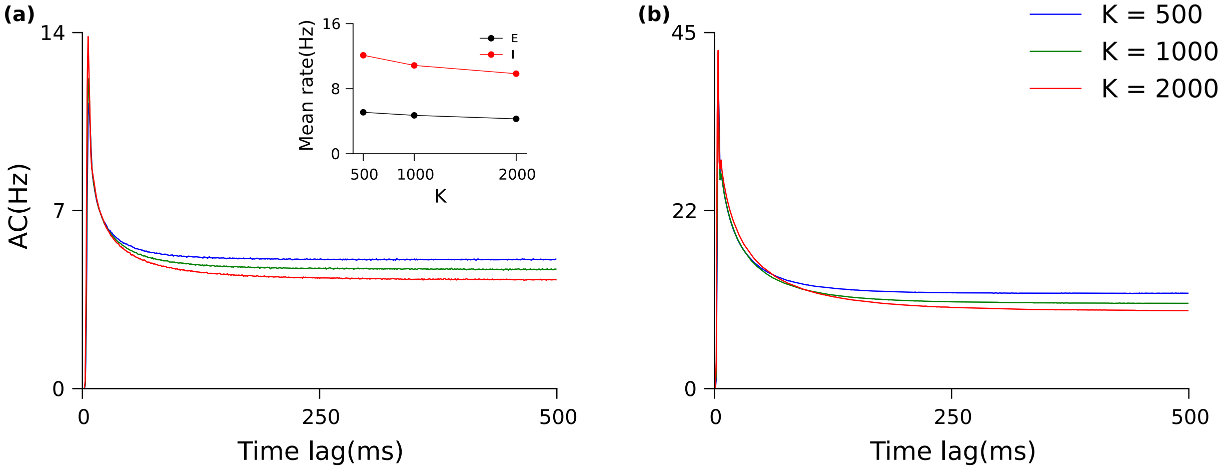

Figure S3. **The slowing down of dynamics is independent of K.** Population averaged autocorrelation functions with excess bidirectionality in the inhibitory population with $p = 0.8$ for (a) excitatory population (b) inhibitory population. Inset shows the small changes in population averaged firing rates with different values of K

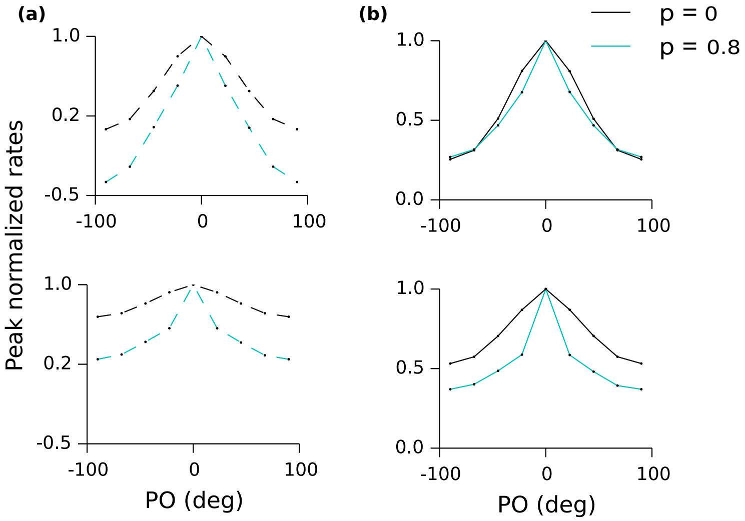

Figure S4. Effect of excess I-I bidirectionality on input and output tuning curves. a: Population average tuning curve of the input. The tuning sharpens for excitatory (top) and inhibitory (bottom) populations when p is increased. b: Population average tuning curve for the response of the neurons. The tuning curve sharpens with p for the inhibitory population (bottom). The effect on the excitatory tuning is negligible (top).
